# Whole-Genome Survey Analyses of Five Goby Species Provide Insights into Their Genetic Evolution and Invasion-Related Genes

**DOI:** 10.3390/ijms25063293

**Published:** 2024-03-14

**Authors:** Siyu Ma, Xiang Zhao, Na Song

**Affiliations:** Key Laboratory of Mariculture, Ocean University of China, Ministry of Education, Qingdao 266003, China; msy05011@163.com (S.M.); zx15965582296@163.com (X.Z.)

**Keywords:** whole-genome survey, Gobiidae, microsatellite, phylogenetic, PSMC

## Abstract

As one of the most abundant groups in marine fish families, Gobiidae fish are important fishery resources in China, and some are also invasive species in certain regions worldwide. However, the phylogenetic relationships of Gobiidae fish remain ambiguous, and the study of their invasion-related genes is still scarce. This study used high-throughput sequencing technology to conduct a whole-genome survey of five Gobiidae fish species: *Acanthogobius flavimanus*, *Acanthogobius stigmothonus*, *Favonigobius gymnauchen*, *Ctenotrypauchen microcephalus*, and *Tridentiger barbatus*. De novo assembly of five fish genomes was performed, and genomic traits were compared through K-mer analysis. Among the five Gobiidae fish genomes, *F. gymnauchen* had the largest genome size (1601.98 Mb) and the highest heterozygosity (1.56%) and repeat rates (59.83%). Phylogenetic studies showed that *A. flavimanus* was most closely linked to *A. stigmothonus*, while Apogonidae and Gobiidae were closely related families. PSMC analysis revealed that *C. microcephalus* experienced a notable population expansion than the other four fish species in the Early Holocene. By using the KOG, GO, and KEGG databases to annotate single-copy genes, the annotated genes of the five fish were mainly classified as “signal transduction mechanisms”, “cellular process”, “cellular anatomical entity”, and “translation”. *Acanthogobius flavimanus*, *A. stigmothonus*, and *T. barbatus* had more genes classified as “response to stimulus” and “localization”, which may have played an important role in their invasive processes. Our study also provides valuable material about Gobiidae fish genomics and genetic evolution.

## 1. Introduction

The family Gobiidae belongs to Perciformes, Gobioidei, and includes 242 genera and over 2000 species [1]. Gobiidae fish are the most abundant group of marine fishes and are extensively distributed in seawater and freshwater, including marine waters, intertidal mudflats, deltas, and rivulets [2,3]. Gobiidae fish are not only predators of benthos but also the main sustenance source for high trophic-level fishes, and some species are commercial fishing targets [4]. Because of the few distinguishable morphological characteristics and the small size of most Gobiidae species, their phylogenetic analysis and morphology identification are still a great challenge [1,5]. Additionally, they generally have significant colonial potential due to rapid reproduction and strong adaptability [6]. Some members of Gobiidae are considered invasive organisms and have a significant effect on native species. For instance, *Rhinogobius giurinus* and *Rhinogobius cliffordpopei* have invaded the Nine Plateau Lakes in Yunnan, China, which has led to the extinction of numerous indigenous fish species [7]. *Acanthogobius flavimanus* and *Tridentiger bifasciatus* have expanded throughout parts of Australia, Europe, and the western coast of the United States [8,9]. In the San Francisco Estuary, *Tridentiger barbatus* has been identified as an invasive species [5]. These invasive Gobiidae species have established populations in non-native habitats by ballast water discharge or accidental releases [8]. These results suggested that these species may have regulatory mechanisms for environmental adaptability. However, the genes related to environmental adaptability in Gobiidae fish are still unexplored.

The genome is vital for studying biological mysteries as it contains all genetic information [5]. Bioinformatics can better quantify and define the genomes of organisms by analyzing and comparing genomic sequences. It can also elucidate the relationships between various organisms [10]. At present, a large amount of morphological data has been accumulated on Gobiidae species [11]. There have also been molecular biology studies of evolutionary and genetic relationships in Gobiidae. For example, the microsatellite characteristics of 19 Gobiidae fish provided powerful support for microsatellite development in Gobiidae [12]. Complete mitogenome assembly in three Gobiidae fish demonstrated that the evolution of mitochondrial genome protein-coding genes (mtDNA PCGs) was crucial to Gobiidae adaptability [13]. Although the Gobiidae family contains more than 2000 species [1], only 18 species’ genomes have been published (https://www.ncbi.nlm.nih.gov/genome/?term=Gobiidae, accessed on 7 March 2024). Genomic information about Gobiidae fish is still limited, which presents significant challenges to exploring the evolutionary processes, genetic diversity, and gene function of Gobiidae fish.

In recent years, next-generation sequencing (NGS) has provided convenient, elaborate, cheap, and full-scale methods for genetic research [14]. High-throughput sequencing is considered a cost-effective technology for obtaining prior knowledge of genomic information [15,16]. Whole-genome survey analysis, based on high-throughput sequencing data, could calculate genome size, repeat ratio, heterozygosity ratio, and GC content [17,18]. Furthermore, NGS technology may help the identification of microsatellites (SSRs), the extraction of mitochondrial genomes and single-copy homologous genes, and the prediction of historical demographic dynamics [19,20]. High-throughput sequencing is considered a time-saving and accessible way to generate genome sequences. With the advancement of sequencing technology, phylogenetic analysis based on mitochondrial genes and single-copy homologous genes has become more accessible. The phylogenetic trees constructed using mitochondrial genomes have shown complex relationships and divergence times among Gadidae species [21]. Using single-copy genes from the whole genome of 10 Gobiidae species for phylogenetic analysis has indicated that evolutionary relationships may be efficiently analyzed [22]. In summary, genome data acquired by NGS have grown to be a valuable method for clarifying the genetic relationships among multiple species. Moreover, compared with conventional methods, NGS is a cheaper and more convenient technology for screening high-polymorphism primers of microsatellites [23]. Thus, there has been increasing attention to developing high-polymorphism microsatellite markers by using NGS data.

To supplement Gobiidae fish genomics information, clarify the genetic evolutionary relationships, and focus on invasion-related genes, we obtained the genomic draft of five Gobiidae fish (*A. flavimanus*, *Acanthogobius stigmothonus*, *Favonigobius gymnauchen*, *Ctenotrypauchen microcephalus,* and *T. barbatus*). Among them, the phylogenetic relationship between *Acanthogobius* and *Favonigobius* has been disputed, and *A. stigmothonus* is usually misclassified as *A. flavimanus* due to their similar morphological characteristics [24]. Moreover, *A. flavimanus* and *T. barbatus* were considered invasive species [5,6,7,8]. This study not only provides new perspectives on classification and evolutionary biology but also furthers genomics research into Gobiidae fish.

## 2. Results

### 2.1. Whole-Genome Sequencing

The raw data generated for *A. flavimanus*, *A. stigmothonus*, *F. gymnauchen*, *C. microcephalus*, and *T. barbatus* amounted to 52.48 Gb, 54.54 Gb, 53.77 Gb, 53.55 Gb, and 52.22 Gb, respectively, through NGS sequencing. Table 1 illustrates the quality statistics of raw data, including the effective rate, error rate, Q20, Q30, and GC content. The sequencing data exhibited an error rate below 1%, and all the Q20 and Q30 values surpassed 95% and 91%, respectively, which affirms the reliability of the sequencing data.

### 2.2. K-mer Analysis

**Figure 1:**
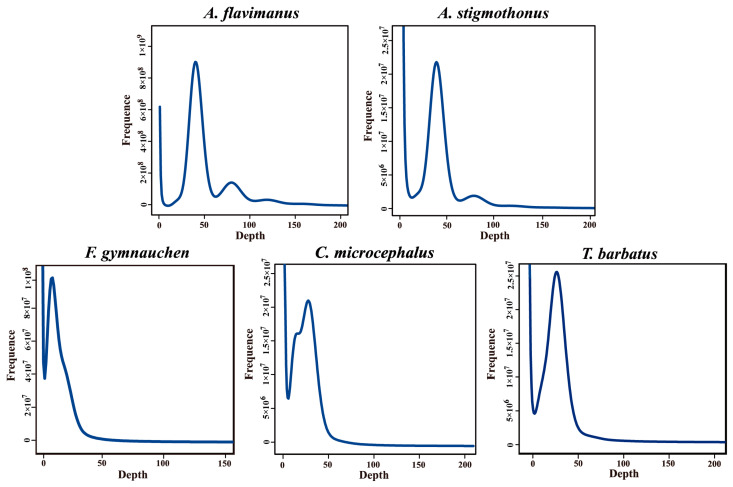
K-mer analysis of five Gobiidae species.

K-mer analyses revealed that the K-mer depth of five species was 39, 39, 11, 34, and 39, respectively, as illustrated in Figure 1. Genome sizes could be calculated by K-mer number and depth. Among the five Gobiidae species, *T. barbatus* exhibited the smallest genome size (808.55 Mb), and *F. gymnauchen* had the largest genome size (1601.98 Mb), as indicated in Table 2. *Favonigobius gymnauchen* displayed higher heterozygosity (1.56%) and repeat rate (59.83%) compared to the other four fish. The heterozygosity rates for the remaining four Gobiidae fish ranged from 0.21% to 0.64%, and repeat rates ranged from 46.90% to 52.89%.

### 2.3. Genome Assembly and GC Content

**Figure 2:**
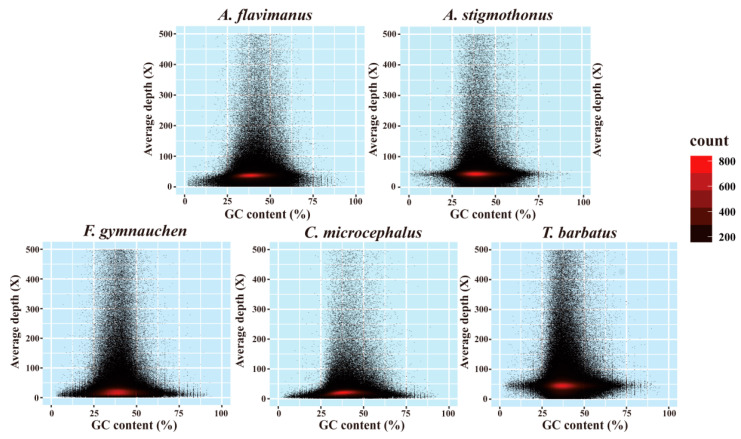
GC content and depth correlation evaluation of five Gobiidae fish.

The filtered clean data were employed for de novo assembly. The contig and scaffold assembled information of the draft genome was presented in Table 3. Among five Gobiidae fish, the total length and total number of *F. gymnauchen* genomes were larger than others, while the N90 length was the shortest. The GC content of *A. flavimanus*, *A. stigmothonus*, *F. gymnauchen*, *C. microcephalus,* and *T. barbatus* were 41.04%, 40.80%, 40.05%, 41.69%, and 39.91%, respectively (Figure 2).

### 2.4. Microsatellite Profile of Five Gobiidae Fish Genomes

**Figure 3:**
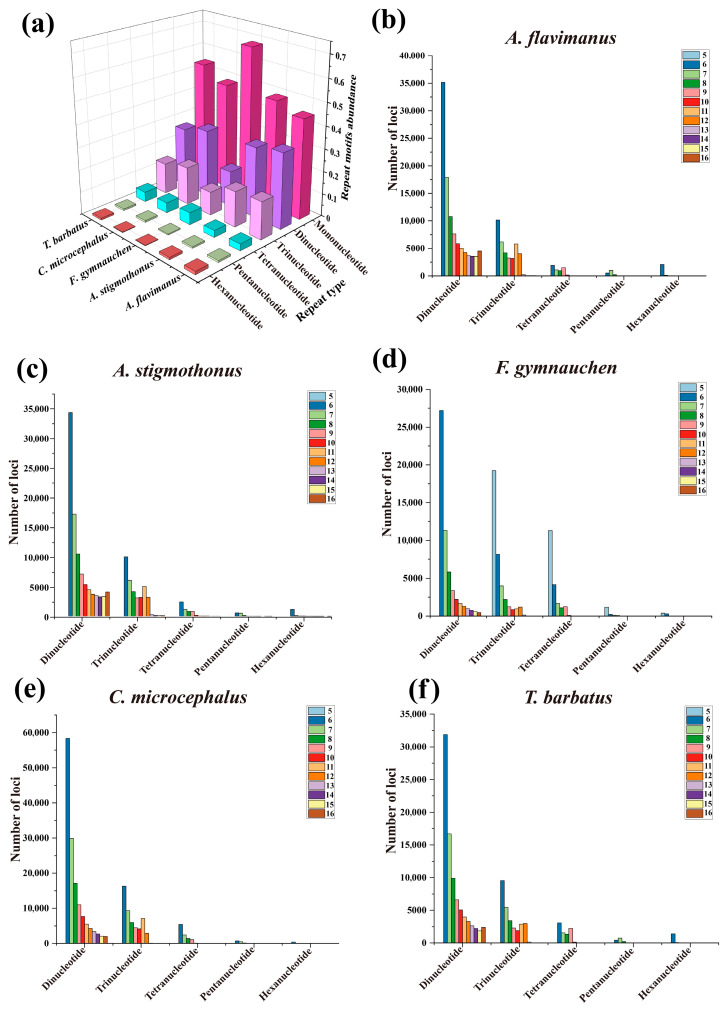
The distribution and abundance of different SSR motifs. (**a**) Percentage distribution of various repeat types. (**b**) The number of SSR motifs in *A. flavimanus*. (**c**) The number of SSR motifs in *A. stigmothonus*. (**d**) The number of SSR motifs in *F. gymnauchen*. (**e**) The number of SSR motifs in *C. microcephalus*. (**f**) The number of SSR motifs in *T. barbatus*.

**Figure 4:**
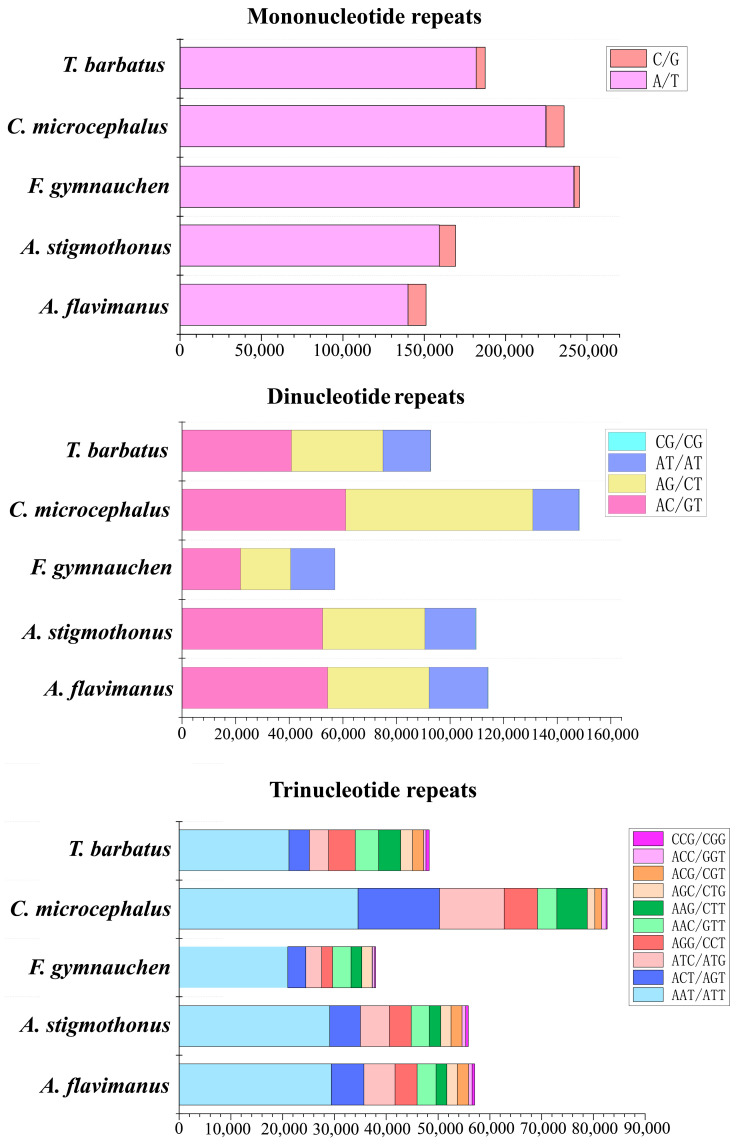
Frequency of mononucleotide, dinucleotide and trinucleotide repeats for five Gobiidae fish.

Through a de novo search for microsatellites, a total of 340,592, 351,606, 362,191, 494,515, and 349,412 microsatellite motifs were identified in the genomes of five Gobiidae fish. Detailed statistics of microsatellites are shown in Table 4. The distribution of microsatellites in *F. gymnauchen* and *C. microcephalus* genomes followed the order of mononucleotide (mono, 47.70–67.76%) > dinucleotide (di, 15.74–29.97%) > trinucleotide (tri, 10.47–16.71%) > tetranucleotide (tetra, 4.62–5.39%) > pentanucleotide (penta, 0.44–0.79%) > hexanucleotide (hexa, 0.19–0.21%). On the other hand, *A. flavimanus*, *A. stigmothonus*, and *T. barbatus* genomes exhibited a distribution mode of mono- (44.37–53.60%) > di- (26.56–33.53%) > tri- (13.82–16.76%) > tetra- (3.07–4.42%) > hexa- (0.70–0.82%) > penta- (0.85–1.44%) (Figure 3a). The number of microsatellite repetitions primarily varied from 5 to 16, with a decline in the number of microsatellites observed as the repeat numbers increased (Figure 3b). Among the motif types of microsatellite repeats in the genomes of five Gobiidae fish, A/T and AAT/ATT were the dominant repeats among the mononucleotide and trinucleotide repeat motifs (Figure 4). Regarding the dinucleotide repeat motif, AC/GT was the most abundant repeat in *A. flavimanus*, *A. stigmothonus*, *F. gymnauchen* and *T. barbatus*, while AG/CT was the most abundant in *C. microcephalus*. Moreover, tetranucleotide, pentanucleotide and hexanucleotide repeats had the fewest motifs in the genomes of five fish, with the repeats having A + T > 50% being the most abundant (Table S1).

### 2.5. Genome Annotation

**Figure 5:**
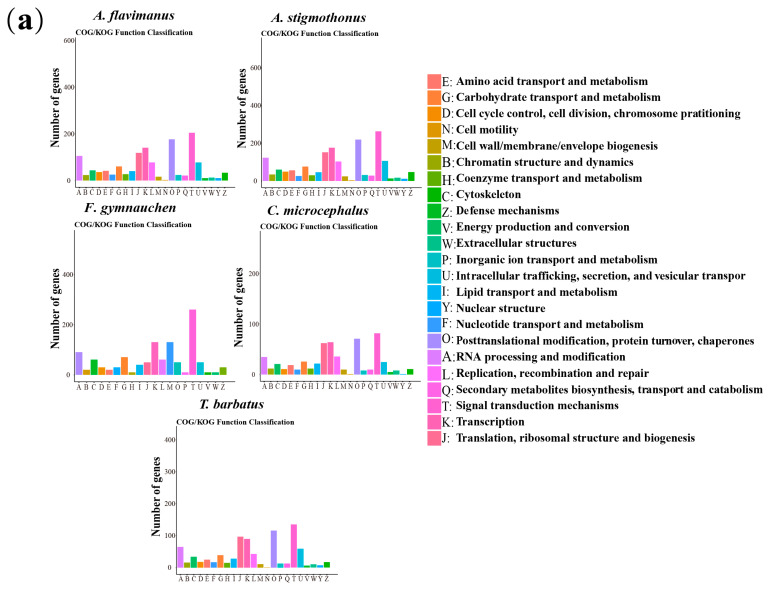

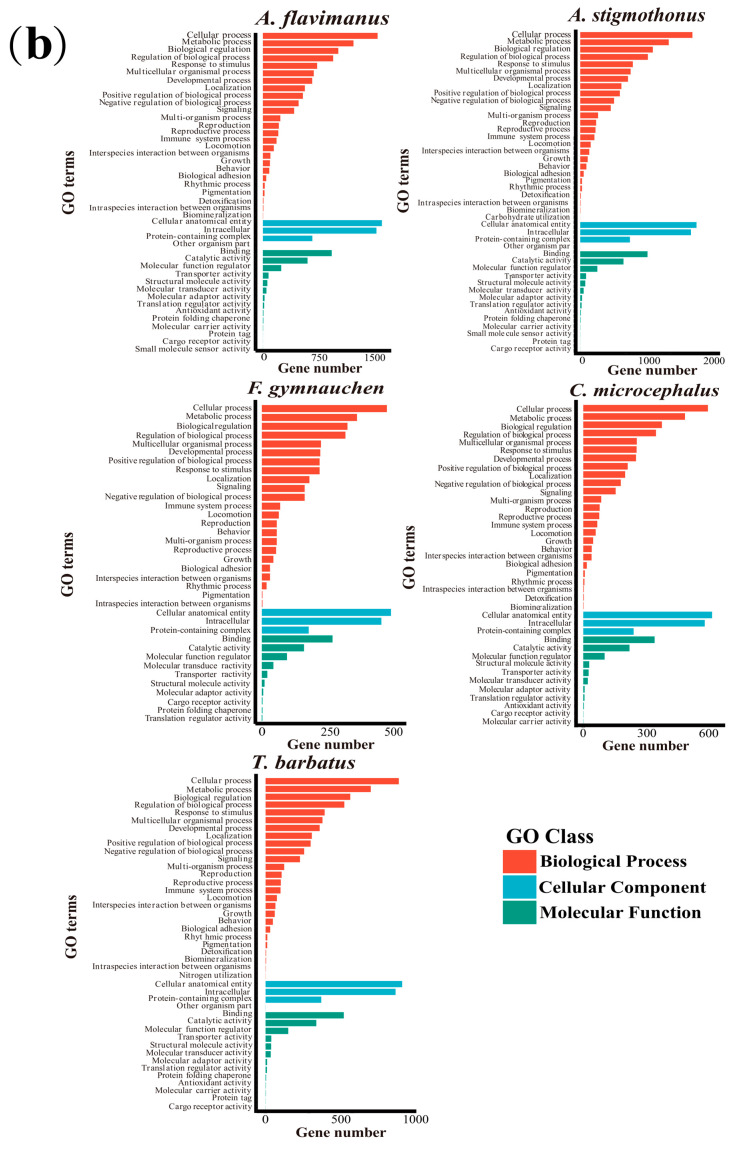

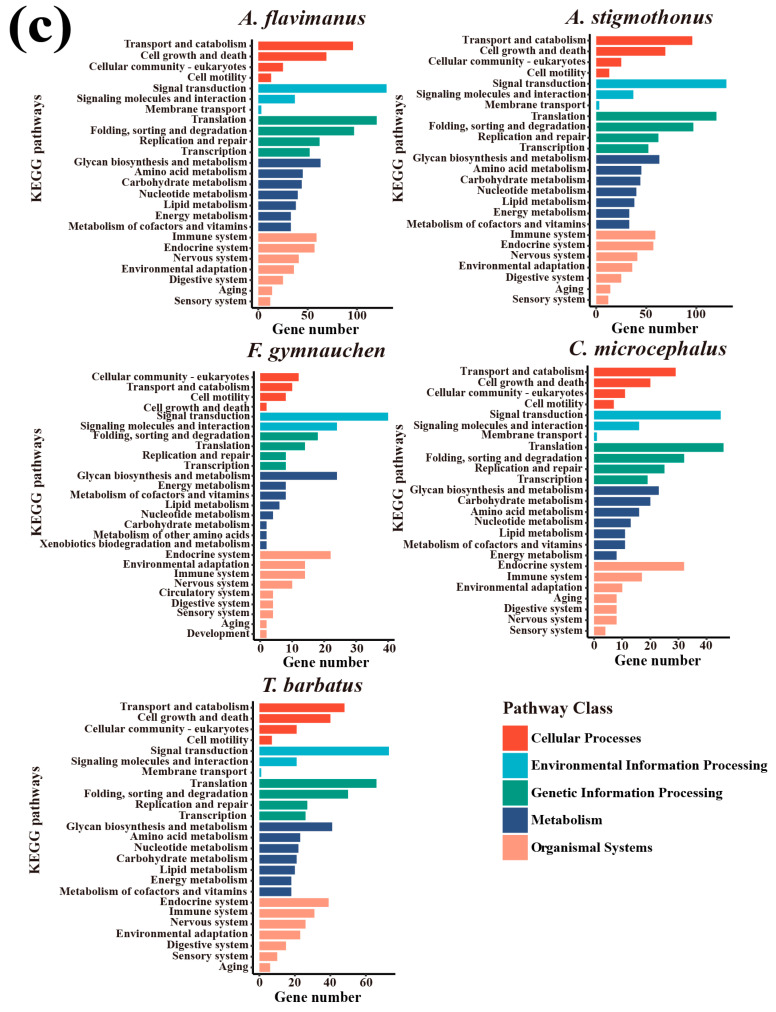
All predicted single-copy genes were aligned by BLAST to three information systems: (**a**) KOG annotation of predicted single-copy genes. (**b**) GO annotation of predicted single-copy genes. (**c**) KEGG annotation of predicted single-copy genes.

**Figure 6:**
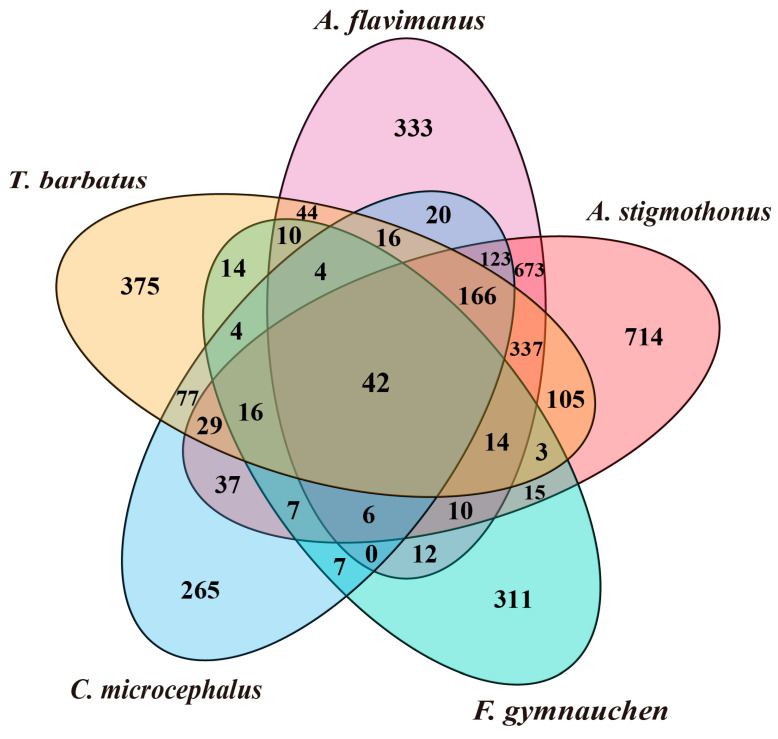
Venn diagram of annotated genes of five Gobiidae fish. Numbers represent the count of overlapping annotated genes.

A total of 1780, 2250, 663, 794, and 1219 single-copy genes from *A. flavimanus*, *A. stigmothonus*, *F. gymnauchen*, *C. microcephalus* and *T. barbatus* were annotated in the KOG database. The statistical map of KOG annotations revealed that the top four annotated genes in the genomes of five Gobiidae fish were classified as “signal transduction mechanisms” “transcription” “translation, ribosomal structure, and biogenesis” and “RNA processing and modification” (Figure 5a). GO functions were classified into three categories, and the GO annotated genes in the genomes of five fish were mainly classified as “cellular process”, “cellular anatomical entity” and “binding”. Additionally, *A. flavimanus*, *A. stigmothonus* and *T. barbatus* had more genes classified as “response to stimulus” and “localization” compared to the other two Gobiidae fish (Figure 5b). KEGG annotation results demonstrated that the genomes of five fish had more genes classified as “signal transduction”, “translation”, “transport and catabolism”, “endocrine system” and “glycan biosynthesis and metabolism” (Figure 5c). Among the annotated genes, 42 overlaps were found to be common among five fish, with *A. flavimanus* and *A. stigmothonus* having the highest number of overlapping genes (673 genes) (Figure 6).

### 2.6. Mitochondrial Genome Assembly and Phylogenetic Analysis

**Figure 7:**
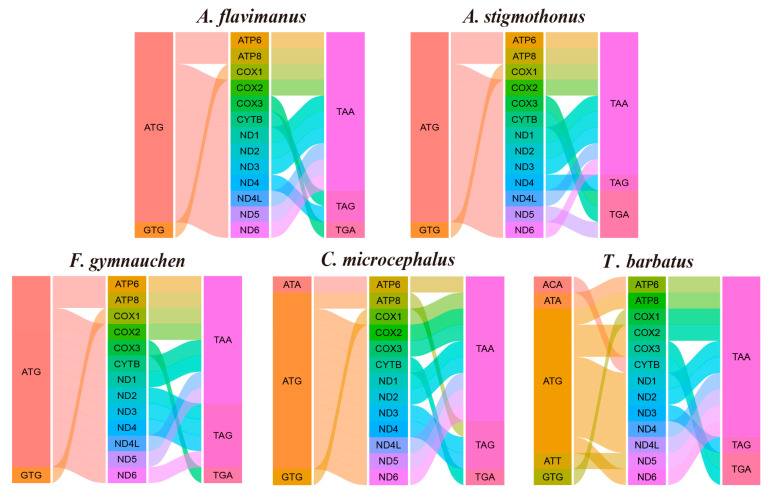
Start and stop codons used in mitochondrial genomes of five Gobiidae fish. Different colors indicate different genes and codons.

Mitochondrial genomes of five Gobiidae fish were formed into closed circular molecules. The total length of *A. flavimanus*, *A. stigmothonus*, *F. gymnauchen*, *C. microcephalus* and *T. barbatus* mitogenome was 16,779 bp, 16,796 bp, 16,621 bp, 16,650 bp and 16,504 bp, respectively. Mitochondrial genomes all included typical 37 genes (13 protein-coding genes, 22 tRNAs, and 2 rRNAs) and 1 control region (Figure S1). ND6 and eight tRNA genes are distributed on the light chain, and the remaining genes are distributed on the heavy chain. Among the 13 protein-coding genes (PCGs), the start codon of five fish has five types, including ATG, GTG, ATA, ACA, and ATT. The termination codon was TAA, TAG, and TGA (Figure 7). Furthermore, we estimated the ratio of nonsynonymous (Ka) and synonymous (Ks) substitutions among the 13 protein-coding genes. The Ka/Ks ratio of each pair and the average Ka/Ks ratio (0.0851) were less than one (Table S2). This indicated that purifying selection acted against changes, resulting in low levels of purifying selection rates.

**Figure 8:**
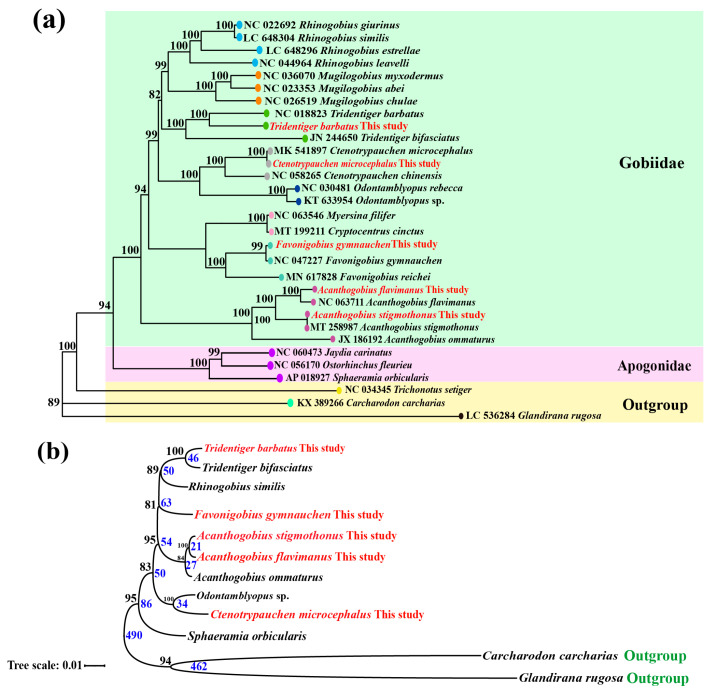
The phylogenetic tree inferred the kinship of 26 species. (**a**) The phylogenetic relationship was constructed using 13 protein-coding genes from 26 species. Species from the same genus are represented by the same background colors. (**b**) Phylogenetic tree of 12 species using single-copy genes. The red lettering means the species in this study. The blue words indicate estimated divergence time of species (million years ago).

To better acquire the phylogenetic relationship, we used the 13 protein-coding genes from 26 species to construct maximum likelihood phylogenetic (Figure 8a). Our study showed that five Gobiidae species were clustered into four clades with high bootstrap values, and the closest kinship is *A. flavimanus* and *A. stigmothonus* which were consistent with traditional morphological classifications. Following fossil calibrations, the divergence time between *A. flavimanus* and *A. stigmothonus* was estimated at 21 million years ago (mya), while the divergence time from *A. ommaturus* was about 27 mya. Furthermore, we found that Apogonidae species had close relationships with Gobiidae species, and their divergence time was estimated at 86 million years ago (mya). The phylogenetic tree constructed using single-copy genes supported the clustering kinship by mitochondrial gene analysis (Figure 8b).

### 2.7. Demographic History

**Figure 9:**
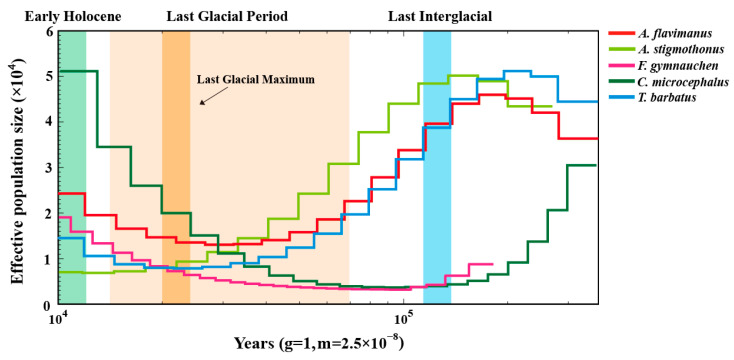
Historical demographic dynamics of five Gobiidae fish.

The PSMC model was employed to analyze the historical demographic dynamics of five Gobiidae fish over the period of 10–200 ka. As depicted in Figure 9, the effective population size (*N_e_*) of all five fish exhibited a continuous decline during the Last Interglacial period. At the beginning of the last glacial period, *F. gymnauchen* and *C. microcephalus* experienced a notable population expansion. The effective population sizes (*N_e_*) of *A. flavimanus*, *A. stigmothonus* and *T. barbatus* were similar during 10–100 ka, with all of them undergoing bottleneck periods in the last glacial period. *N_e_* of *A. flavimanus* and *T. barbatus* began to gradually increase during the last glacial maximum period. Among five Gobiidae fish, *C. microcephalus* demonstrated a significantly larger population size in the Early Holocene.

## 3. Discussion

Gobiidae fish represent the most diversified group among marine fish families, with certain species exhibiting remarkable colonization potential [5,7,8,9]. In this study, the genomes of five Gobiidae fish were investigated using next-generation sequencing technology. The genomic data obtained were then employed to delve into genome characteristics, evolutionary relationships, gene functions, and the dynamic history of populations in these five Gobiidae fish.

### 3.1. Genome Sequencing and Characteristics

The characteristics of the raw data indicated that the effective rate, Q20, and Q30 in all five species exceeded 91%, demonstrating high-quality raw data. Genome size represents the amount of DNA within the gamete genome [25]. K-mer analysis revealed the genome sizes of five Gobiidae fish: 978.96 Mb for *A. flavimanus*, 998.27 Mb for *A. stigmothonus*, 1601.98 Mb for *F. gymnauchen*, 812.88 Mb for *C. microcephalus*, and 808.55 Mb for *T. barbatus*. The genome size of most Osteichthyes fish ranged from 300 Mb to 1 Gb, generally smaller than that of cartilaginous fish genomes [26,27,28]. The reported genome sizes of Gobiidae fish ranged from 679.76 Mb *(Periophthalmus magnuspinnatus*) to 1003.74 Mb (*Neogobius melanostomus*). The genome sizes of two *Acanthogobius* species were relatively large and comparable to *A. ommaturus* (921.49 Mb) [26]. Notably, the genome of *F. gymnauchen* was large, possibly due to a higher repetitive sequence content. The repeat ratio of the genome affects not only genome size and recombination but also the chromosome’s structure [29,30]. If genomic heterozygosity is exorbitant, genome assembly will be difficult [16]. In this study, *F. gymnauchen* exhibited a high heterozygous ratio (1.56%, >1%), which may impact the actual genome assembly. Future studies may benefit from combining second and third-generation sequencing techniques. Moreover, the GC content of five species ranged from 39.91% to 41.69% in our study, within the normal range and suitable for subsequent analysis [31].

### 3.2. Profile of Repeat Sequences in 5 Gobiidae Fish

Genomic SSR markers serve as effective genetic markers and have the advantages of easy detection, multiple allelic polymorphism, and codominant inheritance, which are common markers employed in many studies [32]. Previous research indicated that microsatellite markers also can be used to infer invasion history [33]. However, traditional methods for screening microsatellites are time-consuming and inefficient, while whole-genome survey sequencing presents a promising and cost-effective approach for identifying highly polymorphic microsatellite primer sequences [14,34]. There is limited availability of microsatellite markers in Gobiidae fish, and the existing primers are not universally applicable among species [35]. Until now, there has been no development of microsatellite markers for five Gobiidae fish. In the present study, plenty of candidate microsatellite loci were identified, and the distribution characteristics were described. Mononucleotide repeats were the most common microsatellite loci for five Gobiidae fish, and the number of repeats decreased with the length of the repeat motifs increased. *C. microcephalus* and *F. gymnauchen* exhibited the greatest number of SSRs, which could be attributed to their higher genomic heterozygosity [36]. Despite *C. microcephalus* having a relatively small genome size, it had the highest number of SSRs. This suggested that the number of SSRs is independent of genome size. These findings provide powerful support for further development of microsatellites in Gobiidae fish, and facilitate following studies on species invasion, population genetics, and genetic diversity [37].

### 3.3. Genome Annotation

Single-copy genes, known for their conservation during speciation, play a vital role in regulating diverse life activities and constitute a significant part of functional genomics [38,39]. In this study, we searched single-copy genes within the scaffold draft genomes of five Gobiidae species and mapped them to KOG, GO, and KEGG databases. The results of annotated genes were mainly classified as “signal transduction mechanisms”, “cellular process”, “cellular anatomical entity” and “translation”. It is noteworthy that *A. flavimanus*, *A. stigmothonus* and *T. barbatus* had more genes classified as “response to stimulus” and “localization”. These genes are pivotal in responding to stress, biological stress, and endogenous and extracellular stimuli. They facilitate rapid physiological or behavioral responses to stimuli such as alterations in temperature, light exposure, or biotic interactions [40,41]. Previous research has emphasized the importance of organisms’ responses to environmental stimuli in the context of invasive processes [42]. We speculated that these genes may be a factor in *A. flavimanus* and *T. barbatus* invasion, which promote rapid and appropriate responses to environmental stimuli and could provide a competitive advantage in new habitats [5,8]. Further evidence and research are needed to support this conjecture. Previous studies have demonstrated that the interaction of genes and environmental factors may promote species invasion [43]. Therefore, it is critical to comprehend these genes in organisms’ responses to environmental stress, as well as internal and external stimuli. Furthermore, investigating the expression patterns of these genes can provide valuable insights into their dynamic regulatory mechanisms across various environments [44]. Future research can delve into the intricate functions, regulatory networks, and interactions of these genes, which will enrich our understanding of species’ survival and reproductive strategies.

### 3.4. Overview of Mitochondrial Genome and Phylogenetic Relationship

Mitochondrial genomes were assembled from the scaffold graft genome for five Gobiidae fish. The mitogenomes of *C. microcephalus* and *T. barbatus* were similar to previously reported sequences (No MK_541897, 16,652 bp; No NC_018823, 16,522 bp). However, the mitochondrial length of *A. flavimanus* in this study was longer than the reported sequences (No NC_063711, 16,673 bp), which was attributed to the longer displacement loop (D-loop). It has been reported that D-loops may have mutational changes during evolution, leading to variations in sequence length [45]. We attempted to extract the mitochondrial sequences of *A. stigmothonus* and *F. gymnauchen* using MitoFinder v2 and MitoZ v2.4 software [46,47]. The sequences were longer than previously published sequences (No MT_258987, 16,666 bp; No NC_047227, 16,480 bp), mainly due to the extended length of the 16S rRNA genes. Because of the resilience of 16S rRNA genes to mutation, we speculated that erroneous fragments might have been added into 16S rRNA during high-throughput sequencing and assembly. With the rapid advancement of NGS technology, mitochondrial genome sequences have become more accessible. Currently, in the literature about extracting the complete mitochondrial genome, NGS technology has gradually replaced the conventional method (https://www.tandfonline.com/loi/tmdn20, accessed on 20 February 2024). NGS technology has emerged as a powerful tool for studying evolutionary biology and phylogenetics.

The phylogenetic analysis using 13 protein-coding genes revealed that five fish were clustered into four clades with high bootstrap values. *Ctenotrypauchen* and *Odontamblyopus* were clustered into one clade within Amblyopinae, characterized by a continuous dorsal fin [48,49]. *A. stigmothonus* and *A. flavimanus* were identified as the two most recently diverged species in Gobiidae, and they had the closest relationship, which was aligned with morphological classification results [24,50]. It supported that their divergence did not occur simultaneously, despite their sequences being extremely similar and often misidentified. Furthermore, *Favonigobius* and *Acanthogobius* were divided into two branches, which was inconsistent with the results of 12S rRNA sequences [51]. We speculated that the limitations imposed by the 12S rRNA of mitochondrion on exploring evolutionary relationships among species. This phylogenetic tree also supported the conclusion that Apogonidae species had close relationships with Gobiidae species [48,52]. The divergence time between Apogonidae and Gobiidae species was estimated to be 86 mya, which consistently aligned with previous studies utilizing time plots [53]. Furthermore, single-copy genes exhibited high conservation during evolution and could provide more accurate phylogenetic relationships [24,54]. The phylogenetic tree constructed by them also confirmed the precision of 13 protein-coding genes.

### 3.5. Demographic Analysis

Historical demographic dynamics can explore the impact of external factors on species distribution [55]. In this study, PSMC analyses of five Gobiidae species showed that their effective population size had a continuous decline during the last interglacial period. This was a relatively warm geological period compared to preceding glacial periods, and some populations may migrate to more suitable habitats [56]. During the Pleistocene Glacial Epoch, *A. flavimanus* and *T. barbatus* experienced a bottleneck period and occurred a gradual recovery after the last glacial maximum. This finding was aligned with *T. bifasciatus* [22]. In contrast, the population size of *F. gymnauchen* and *C. microcephalus* exhibited a continuing population expansion, while *A. stigmothonus* experienced a continuing decrease. During the Pleistocene Glacial Epoch, the sea level decreased by approximately 100 meters, destroying coastal fish habitats. However, the persistence of water sources unaffected by glaciers may have facilitated population increases. In summary, the combined effects of changes in sea area and climate change during this period may have a profound impact on the abundance and distribution of marine life [57,58]. It is noteworthy that the population size of *C. microcephalus* was significantly larger than the other four fish during the Early Holocene, suggesting a potential increase in genetic diversity. Genetic diversity plays a crucial role in influencing the abundance and distribution of species, and the higher genetic diversity of *C. microcephalus* may be related to its abundance of resources, which can be found in the reports of dominant species [4,59,60].

## 4. Materials and Methods

### 4.1. Ethics Statement

This study was conducted strictly with Chinese Animal Management Regulations and animal care quality management requirements of the Ocean University of China.

### 4.2. Sample Preparation and Genome Sequencing

Gobiidae fish samples were collected from the coastal seas of China (Table 5). After morphologically identifying, the muscle tissue of five fish was extracted and preserved in 95% alcohol. The remaining tissue samples were rapidly refrigerated at −80 °C. We extracted DNA from one sample of each species. The phenol-chloroform approach was employed to extract genomic DNA. This method combined cell lysate with phenol, and then separated the aqueous and organic phases. DNA remained in the aqueous phase, while proteins and lipids partitioned into the organic phase. After using chloroform for further purification, DNA was precipitated in ethanol and the ethanol was decanted. Subsequently, the DNA-containing pellet was air-dried and then mixed with 50 μL nuclease-free water. The DNA content was measured with a NanoDrop 2000 Spectrophotometer (Waltham, MA, USA). Then, DNA barcoding technology (Cytochrome Oxidase Subunit I gene and 12S ribosomal RNA gene) was employed for species identification.

The random DNA fragments were obtained using a Covaris ultrasonic crusher (Covaris, Woburn, MA, USA). The construction of the whole library entailed several sequential steps to prepare the DNA fragments for sequencing. Initially, the DNA fragments underwent end repair, trimming any damaged or overhanging ends. Subsequently, adapter molecules were ligated to the repaired DNA fragments’ ends. Following adapter ligation, the library was purified to eliminate any unligated adapters or other contaminants. Finally, Polymerase Chain Reaction (PCR) amplification was conducted to amplify the DNA fragments, enriching the library for sequencing. The prepared library was sequenced using the Illumina HiSeq2500 platform (San Diego, CA, USA), employing synthetic sequencing (SBS) technology. Novogene Co., Ltd. (Beijing, China) constructed the library and performed sequencing.

### 4.3. Analysis of K-mer and GC Content

The impact of bias in GC content and sequencing errors was reduced by Fastp v0.20.1 and 50X sequencing depth [61]. A total of 10,000 filtered readings were selected for comparison with NCBI’s nucleotide (NT) database, which could find the related species. K-mer analysis was performed using Jellyfish v2.0 and GenomeScope v2.0 software to collect information about genome length, heterozygosity, repeat content and genome size [62,63]. Meanwhile, clean reads were assembled into contigs and scaffolds with SOAPdenovo v2.01 software [31]. Scaffold sequences were constructed using paired-end information from contigs, and assembly quality was assessed using Busco v5 software [64]. Furthermore, the sequencing depth and GC content of five fish were evaluated to identify the sequencing bias. The average depth of GC sequencing was calculated using 10 kb non-overlapping sliding windows across the assembled sequences [18]. General graphing and statistics were drafted by R-Studio v4.0 [65].

### 4.4. Microsatellite Identification

The MIcroSAtellite (MISA) v2.1.0 software was employed to identify microsatellite motifs in the whole genome [66]. The size of microsatellite motifs ranged from one to six nucleotides, and criteria were set at 10, 6, 5, 5, 5, and 5, respectively. Subsequently, graphs were generated using Origin 2021 (https://www.originlab.com/2021, accessed on 20 October 2023).

### 4.5. Search of Single-Copy Homologous Genes and Functional Annotation

The single-copy homologous genes of five Gobiidae fish were identified using Busco v5 software [64]. Moreover, the whole gnomes of seven other fish species (*Tridentiger bifasciatus*: PRJNA825009; *Rhinogobius similis*: GCA_019453435.1; *Acanthogobius ommaturus*: SRR1749713; *Odontamblyopus* sp.: PRJNA834853; *Sphaeramia orbicularis*: GCA_902148855.1; *Carcharodon carcharias*: GCA_017639455.1; *Glandirana rugosa*: GCA_018402905.1) were downloaded from the NCBI (http://www.ncbi.nlm.nih.gov/genome/, accessed on 10 November 2023) database, and used for multiple sequence alignment to construct a phylogenetic tree by OrthoFinder v2.5.2 software [67]. Divergence time was calculated and calibrated based on MCMCTree v4.9 software and the TreeTime database [68,69]. Then, all identified single-copy genes in five Gobiidae fish were annotated using eggNOG-mapper v2.1.5 software [70]. Gene annotation of the Eukaryotic Orthologous Group (KOG), Gene Ontology (GO) and Kyoto Encyclopedia of Genes and Genomes (KEGG) was performed using the eggnog database. The results of gene annotation were visualized using HIPLOT PRO (https://hiplot.com.cn/cloud-tool/drawing-tool/list, accessed on 17 November 2023). A Venn diagram was generated using TBtools v1.098 software (https://github.com/CJ-Chen/TBtools, accessed on 19 November 2023).

### 4.6. Mitochondrial DNA Assembly and Phylogenetic Research

The filtered clean data were assembled to generate complete mitogenome sequences by MitoFinder v2 software [47]. The mitochondrial reference file was downloaded from NCBI with the ID JX186196.1. The assembled sequences were annotated using the mitofish website (http://mitofish.aori.u-tokyo.ac.jp/, accessed on 25 November 2023) to create a circular diagram of the mitochondrial genome.

To analyze the phylogenetic relationship among five Gobiidae fish, the 13 protein-coding sequences in mitochondrion genes of 26 species were collected from the NCBI database (Table S3). The combined sequences were aligned using the Muscle technique in MEGA version X software [71]. The evolutionary relationship was generated using the maximum likelihood (ML) algorithm, providing an accurate analysis of kinship. Additionally, the Ka/Ks ratio (nonsynonymous substitutions/synonymous substitutions) of 13 protein-coding genes in five fish was calculated using MEGAX software [71]. Ultimately, the tree was visualized using the Interactive Tree of Life (iTOL) website, available at https://itol.embl.de/, accessed on 13 December 2023 [72].

### 4.7. Demography History Analysis

The PSMC was employed to estimate the historical demographic dynamics of five fish species across time [73]. The PSMC input file was generated with a quality threshold of 20 (-q) using fq2psmcfa. PSMC analysis used default parameters (-N25-t15-r5-p “4 + 25 × 2 + 4 + 6”) to deduce variations in effective population size (*N_e_*) over time [73]. Results were plotted on different scales of the x-axis with one-year generation time. The mutation rate per generation was set at 2.5 × 10^−8^ using the calculated annual mutation rate per site. Subsequently, PSMC trajectories of five species were plotted together.

## 5. Conclusions

In the present study, the phylogenetic tree revealed that *A. flavimanus* and *A. stigmothonus* have the closest kinship, and *F. gymnauchen* does not belong to *Acanthogobius* contrary to previous reports. Furthermore, we found that Apogonidae species had close relationships with Gobiidae species. The divergence time between Apogonidae and Gobiidae species was estimated to be 86 mya. *A. flavimanus* and *T. barbatus* had more genes classified as “response to stimulus” and “localization”, suggesting their potential importance in invasion biology. PSMC analyses of five Gobiidae species showed that their effective population size had a continuous decline during the last interglacial period. Additionally, plenty of candidate microsatellite loci were identified and provided powerful support for further development of microsatellites in Gobiidae fish. Overall, this paper not only further studies population genetics but also serves as a valuable reference for future studies on gene function and evolution within Gobiidae species.

## Figures and Tables

**Table 1:** Sampling details for five Gobiidae fish.

Species	Raw Base (Gb)	Effective Rate (%)	Error Rate (%)	Q20 (%)	Q30 (%)	GC Content (%)
*A. flavimanus*	52.48	99.51	0.04	96.31	91.18	41.04
*A. stigmothonus*	54.54	99.54	0.04	96.78	92.22	40.80
*F. gymnauchen*	53.77	99.18	0.05	96.83	91.19	40.05
*C. microcephalus*	53.55	96.87	0.03	96.43	91.42	41.69
*T. barbatus*	52.22	98.52	0.03	96.58	91.61	39.91

**Table 2:** Data statistics and analysis of K-mer for five Gobiidae species.

Species	K-mer Number	Genome Size (Mb)	Heterozygous Ratio (%)	Repeat Ratio (%)
*A. flavimanus*	38,806,743,595	978.96	0.21	50.94
*A. stigmothonus*	39,715,938,956	998.27	0.24	50.52
*F. gymnauchen*	44,941,714,426	1601.98	1.56	59.83
*C. microcephalus*	47,124,301,675	812.88	0.64	52.89
*T. barbatus*	45,948,893,647	808.55	0.56	46.90

**Table 3:** Statistics on assembled genomes of five Gobiidae fish.

Species	Assembly Level	Total Length (bp)	Total Number	Max Length (bp)	N50 Length (bp)	N90 Length (bp)
*A. flavimanus*	Contig	750,612,336	766,615	72,830	3318	382
	Scaffold	764,365,216	612,519	78,798	4596	542
*A. stigmothonus*	Contig	801,994,264	783,027	142,602	6066	301
	Scaffold	817,012,736	631,430	230,933	12,605	508
*F. gymnauchen*	Contig	1,538,128,271	7,370,457	75,210	2120	122
	Scaffold	1,558,061,154	6,001,135	90,310	3120	129
*C. microcephalus*	Contig	789,064,495	2,896,033	22,967	4431	150
	Scaffold	782,790,775	2,117,910	24,126	8719	335
*T. barbatus*	Contig	811,858,718	1,790,899	35,267	2116	245
	Scaffold	805,390,951	1,216,192	35,267	2990	307

**Table 4:** Statistics of microsatellite motifs for five Gobiidae fish.

	*A. flavimanus*	*A. stigmothonus*	*F. gymnauchen*	*C. microcephalus*	*T. barbatus*
Total amount of sequences assessed	612,519	631,430	6,510,510	2,117,910	1,216,192
Total size of assessed sequences (bp)	764,365,216	817,012,736	1,653,145,701	806,337,820	817,882,156
Total identified SSRs	340,592	351,606	362,191	494,515	349,412
Number of sequences containing SSRs	163,241	106,981	337,951	371,928	226,596
Number of sequences with multiple SSRs	72,809	55,926	21,999	81,266	72,335
Number of SSRs involved in compound synthesis	57,550	62,289	15,217	73,846	41,068

**Table 5:** Sampling information of five species in Gobiidae fish.

Genus	Species	Sampling Time	Sampling Site	Sample Quantity Obtained	Sample Quantity Used
*Acanthogobius*	*Acanthogobius flavimanus*	26 November 2018	Qingdao, Yellow Sea of China	24	1
	*Acanthogobius stigmothonus*	2 September 2020	Beihai, South China Sea	18	1
*Favonigobius*	*Favonigobius gymnauchen*	20 November 2019	Qingdao, Yellow Sea of China	1	1
*Ctenotrypauchen*	*Ctenotrypauchen microcephalus*	12 April 2022	Rizhao, Yellow Sea of China	12	1
*Tridentiger*	*Tridentiger barbatus*	20 November 2021	Yantai, Bohai Sea of China	20	1

## Data Availability

The whole-genome sequencing data has been uploaded to the NCBI with No. PRJNA1037321; PRJNA1037349; PRJNA1040458; PRJNA1041613; PRJNA1041612.

## Supplementary Materials

The following supporting information can be downloaded at: https://www.mdpi.com/article/10.3390/ijms25063293/s1.
