# Supplementary material for: Whole-Genome Survey Analyses of Five Goby Species Provide Insights into Their Genetic Evolution and Invasion-Related Genes
Source: Int J Mol Sci. 2024 Mar 14;25(6):3293. doi: 10.3390/ijms25063293 (PMC10970681; doi:10.3390/ijms25063293)
Supplement: Supplementary file 1 [file ijms-25-03293-s001.zip › ijms-2856156-supplementary.pdf]

Table S1. The most frequent tetra-, Penta- and hexanucleotide repeats in different genomic sequence of 5 Gobiidae fish (Only the most frequent 10 motifs of different type are show here. The number of motifs is in parenthesis)

	A. flavimanus	*A. stigmothonus*	*F. gymnauchen*	*C. microcephalus*	*T. barbatus*
Tetra	AAAT/ATTT (2351)	AAAT/ATTT (2369)	AATC/ATTG (4589)	AAAT/ATTT (8542)	AAAT/ATTT (3505)
	AATG/ATTC (1330)	AATG/ATTC (1344)	AAAT/ATTT (4144)	AAAC/GTTT (2578)	AAAC/GTTT (1582)
	ATCC/ATGG (1142)	AGAT/ATCT (1107)	AGAT/ATCT (3274)	AATC/ATTG (1928)	AATG/ATTC (1446)
	AGAT/ATCT (1060)	ATCC/ATGG (976)	ATCC/ATGG (1562)	AAAG/CTTT (1650)	AGAT/ATCT (1241)
	ACAT/ATGT (875)	ACAT/ATGT (882)	AAAC/GTTT (1313)	AGAT/ATCT (1615)	ATCC/ATGG (1198)
	ACGC/CGTG (850)	ACGC/CGTG (851)	AAGT/ACTT (1089)	ACAT/ATGT (1363)	ACGC/CGTG (1087)
	AAAC/GTTT (676)	AAAG/CTTT (704)	ACAT/ATGT (778)	ATCC/ATGG (1350)	AAAG/CTTT (992)
	AAAG/CTTT (482)	AAAC/GTTT (672)	AATG/ATTC (768)	AATG/ATTC (1222)	ACAT/ATGT (808)
	ACAG/CTGT (296)	ACAG/CTGT (385)	AAAG/CTTT (502)	ACAG/CTGT (380)	AAGG/CCTT (683)
	AAGT/ACTT (255)	AATC/ATTG (226)	ACAG/CTGT (354)	AGGG/CCCT (366)	AATC/ATTG (676)
Penta	AAAAT/ATTTT (702)	AAAAT/ATTTT (487)	AAGAT/ATCTT (254)	AAGAT/ATCTT (1198)	AAAAT/ATTTT (399)
	AATTC/AATTG (366)	AATTC/AATTG (438)	AATAG/ATTCT (239)	AAAAT/ATTTT (513)	AATTC/AATTG (211)
	AATAT/ATATT (305)	AATAT/ATATT (258)	ACTAT/AGTAT (126)	AATAT/ATATT (446)	AATAT/ATATT (194)
	ACTAT/AGTAT (145)	AAGGC/CCTTG (137)	AAACT/AGTTT (112)	AATAG/ATTCT (204)	AAACT/AGTTT (172)
	AAATT/AATTT (137)	AGATG/ATCTC (120)	AATTC/AATTG (107)	AAACT/AGTTT (180)	AAATT/AATTT (132)
	AAGAT/ATCTT (104)	AAGAT/ATCTT (107)	AATAT/ATATT (83)	ACTAT/AGTAT (150)	AAATC/ATTTG(125)
	AAACT/AGTTT (96)	ACTAT/AGTAT (105)	AAAAT/ATTTT (82)	AAAAC/GTTTT (146)	ACTAT/AGTAT (121)
	AAGGC/CCTTG (67)	AAATT/AATTT (91)	AAGGC/CCTTG (82)	AAATT/AATTT (138)	AAGAT/ATCTT (93)
	AAAGT/ACTTT (61)	AAACT/AGTTT (81)	AATAC/ATTGT (78)	AATCT/AGATT (89)	AATAC/ATTGT (88)
	ACTCT/AGAGT (58)	AATAC/ATTGT (55)	AAATC/ATTTG (73)	AGAGG/CCTCT (85)	AAAAC/GTTTT (81)
Hexa	AATCAG/ATTCTG (1294)	AATCAG/ATTCTG (1044)	ACCAGG/CCTGGT (133)	AACCCT/AGGGTT (94)	ACCAGG/CCTGGT (442)
	AATGCT/AGCATT (362)	AATGCT/AGCATT (365)	ACCTGG/AGGTCC (62)	ACCAGG/CCTGGT (67)	ACCTGG/AGGTCC (207)
	ACCAGG/CCTGGT (359)	ACCAGG/CCTGGT (269)	AACCCT/AGGGTT (36)	AATCAT/ATGATT (51)	AATCAG/ATTCTG (154)
	AACCCT/AGGGTT (244)	AACCCT/AGGGTT (155)	ACTGCT/AGCAGT (27)	AAAAAT/ATTTTT (46)	AGATAT/ATATCT (113)
	ACACGC/CGTGTG (241)	ACACGC/CGTGTG (146)	AGATAT/ATATCT (22)	AAGTCT/ACTTAG (42)	AAATAT/ATATTT (104)
	AAAAAT/ATTTTT (208)	AAAAAT/ATTTTT (143)	AAGTCT/ACTTAG (21)	AGATAT/ATATCT (32)	AAAAAT/ATTTTT (102)
	ACCTGG/AGGTCC (92)	ACCTGG/AGGTCC (87)	AATCTG/AGATTC (21)	AATACT/AGTATT (31)	AACCCT/AGGGTT (98)
	AGATAT/ATATCT (76)	ACTGCT/AGCAGT (55)	AGCTCC/AGCTGG (20)	AAGATT/AATCTT (30)	AATGCT/AGCATT (84)
	AATCAT/ATGATT (62)	AGATAT/ATATCT (54)	AACCAG/CTGGTT (18)	ACTGCT/AGCAGT (29)	ACACGC/CGTGTG (83)
	ACTGCT/AGCAGT (62)	AAATAT/ATATTT (36)	AATGCT/AGCATT (15)	AAAATT/AATTTT (28)	ACATAT/ATATGT (71)

Table S2. Ka/Ks of 13 protein-coding genes from 5 fish.

Gene_name	Ka/Ks	*A. flavimanus*	*A. stigmothonus*	*F. gymnauchen*	*C. microcephalus*	*T. barbatus*
COX1	*A. flavimanus*					
	A. stigmothonus	0.023267394				
	F. gymnauchen	0.019969707	0.026790077			
	C. microcephalus	0.030522551	0.032104725	0.034345371		
	T. barbatus	0.02704615	0.030013127	0.022475565	0.0270889	
COX2	*A. flavimanus*					
	A. stigmothonus	0.060606455				
	F. gymnauchen	0.07265503	0.063022154			
	C. microcephalus	0.045728394	0.056603772	0.028153836		
	T. barbatus	0.063853972	0.057404121	0.02105691	0.030771063	
COX3	*A. flavimanus*					
	A. stigmothonus	0.033830438				
	F. gymnauchen	0.021139901	0.076781223			
	C. microcephalus	0.059251483	0.050002446	0.035756256		
	T. barbatus	0.052739352	0.052165389	0.037136786	0.062822879	
CYTB	*A. flavimanus*					
	A. stigmothonus	0.025686321				
	F. gymnauchen	0.036689369	0.038418619			
	C. microcephalus	0.061464298	0.054628561	0.052300792		
	T. barbatus	0.057564782	0.042613161	0.036627495	0.048154799	
ND1	*A. flavimanus*					
	A. stigmothonus	0.02776181				
	F. gymnauchen	0.042805135	0.056486778			
	C. microcephalus	0.034195739	0.046657988	0.024918047		
	T. barbatus	0.037594527	0.052545404	0.039143047	0.044797533	
ND2	*A. flavimanus*					
	A. stigmothonus	0.086301242				
	F. gymnauchen	0.149257814	0.177782857			
	C. microcephalus	0.151264618	0.175352229	0.084774699		
	T. barbatus	0.197704054	0.201940766	0.102867049	0.120892388	
ND3	*A. flavimanus*					
	A. stigmothonus	0.082872491				
	F. gymnauchen	0.035515687	0.069570894			
	C. microcephalus	0.080790481	0.069202102	0.058870527		
	T. barbatus	0.0846185	0.047938153	0.072536516	0.046622086	
ND4	*A. flavimanus*					
	A. stigmothonus	0.115234827				
	F. gymnauchen	0.085850961	0.112414142			

	C. microcephalus	0.0681671	0.104809453	0.068683408	
	T. barbatus	0.062161757	0.096923822	0.097492652	0.085264998
ND4L	*A. flavimanus*				
	A. stigmothonus	0.071899326			
	F. gymnauchen	0.076412678	0.078452457		
	C. microcephalus	0.056133214	0.096154826	0.031457905	
	T. barbatus	0.037656989	0.088976004	0.064726868	0.035591003
ND5	*A. flavimanus*				
	A. stigmothonus	0.088664862			
	F. gymnauchen	0.06846615	0.032898989		
	C. microcephalus	0.100725623	0.070356146	0.035286455	
	T. barbatus	0.068167988	0.027858752	0.056156758	0.699094587
ND6	*A. flavimanus*				
	A. stigmothonus	0.055874729			
	F. gymnauchen	0.122100109	0.19712097		
	C. microcephalus	0.156756248	0.127864651	0.028988908	
	T. barbatus	0.15253861	0.083960605	0.150764474	0.122057646
ATP6	*A. flavimanus*				
	A. stigmothonus	0.058582787			
	F. gymnauchen	0.141045084	0.130554994		
	C. microcephalus	0.090274589	0.113409247	0.094115449	
	T. barbatus	0.103288785	0.133848575	0.079987528	0.089622561
ATP8	*A. flavimanus*				
	A. stigmothonus	0.08179913			
	F. gymnauchen	0.06769897	0.187353176		
	C. microcephalus	0.239273268	0.467495858	0.173938038	
	T. barbatus	0.02157578	0.224273504	0.174205541	0.200117202

Table S3. Data source of the 26 Gobiidae fishes studied in this study.

Family	Genus	Species	Source
Gobiidae	*Acanthogobius*	*Acanthogobius flavimanus*	in this study
		Acanthogobius flavimanus	NC_063711
		Acanthogobius stigmothonus	in this study
		Acanthogobius stigmothonus	MT258987
		Acanthogobius ommaturus	JX186192
	Ctenotrypauchen	*Ctenotrypauchen microcephalus*	in this study
		Ctenotrypauchen microcephalus	MK541897.1
		Ctenotrypauchen chinensis	NC_058265
	Odontamblyopus	*Odontamblyopus rebecca*	NC_030481
		Odontamblyopus sp.	KT633954
	Favonigobius	*Favonigobius gymnauchen*	in this study
		Favonigobius gymnauchen	NC_047227
		Favonigobius reichei	MN617828
	Tridentiger	*Tridentiger barbatus*	in this study
		Tridentiger barbatus	NC_018823
		Tridentiger bifasciatus	JN244650
		Myersina filifer	NC_063546
		Cryptocentrus cinctus	MT199211
	Mugilogobius	*Mugilogobius chulae*	NC_026519
		Mugilogobius myxodermus	NC_036070
		Mugilogobius abei	NC_023353
	Rhinogobius	*Rhinogobius leavelli*	NC_044964
		Rhinogobius giurinus	NC_022692
		Rhinogobius estrellae	LC648296
		Rhinogobius similis	LC648304
Trichonotidae	*Trichonotus*	*Trichonotus setiger*	NC_034345
Apogonidae	*Ostorhinchus*	*Ostorhinchus fleurieu*	NC_056170
	Jaydia	*Jaydia carinatus*	NC_060473
	Sphaeramia	*Sphaeramia orbicularis*	AP018927
Isuridae	*Carcharodon*	*Carcharodon carcharias*	KX389266
Ranidae	*Glandirana*	*Glandirana rugosa*	LC536284

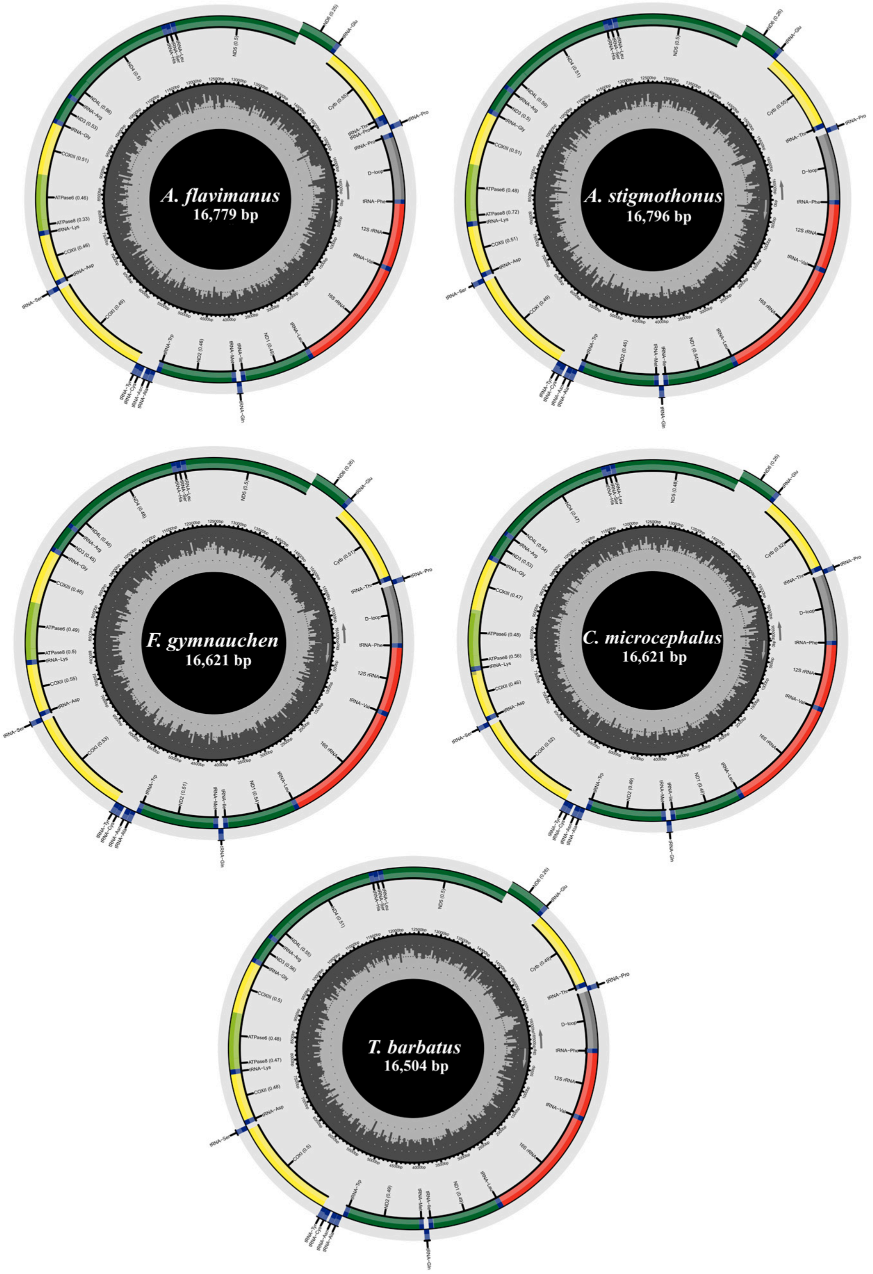

Figure S1. The structure circle diagram of 5 mitochondrial genomes.
